# *EHP* Papers of the Year, 2008

**DOI:** 10.1289/ehp.11684

**Published:** 2008-06

**Authors:** Hugh A. Tilson

**Affiliations:** Editor-in-Chief, *EHP* E-mail: tilsonha@niehs.nih.gov

Authors are often asked about the impact of their research on their fields or disciplines. Likewise, editors often wonder about the impact of articles published in their journals. In an effort to reinforce the high quality of articles published in *EHP*, reveal trends in emerging research themes, and track the impact of groundbreaking research, we will henceforth, on an annual basis, identify the five papers published over the preceding 60 months that have been cited most frequently in the environmental health sciences literature. This month we are pleased to announce the *EHP* Papers of the Year for 2008.

The most frequently cited article for 2008 is “Nanotoxicology: An Emerging Discipline Evolving from Studies of Ultrafine Particles” by Günter Oberdörster, Eva Oberdörster, and Jan Oberdörster. This seminal review, published in July 2005 (Environ Health Perspect 113:823–839), has been cited an average of 62.5 times per year since its publication. The article emphasized that research on ambient ultrafine particles provides the foundation for studying and understanding the biokinetics and toxicologic potential of engineered nanomaterials, including particles, tubes, shells, and quantum dots. It also helped establish that greater surface area per mass predisposes nanoparticles to be more biologically active than larger-sized particles of the same chemistry; moreover, particle surface areas and number seem to be reasonably good predictors for nanoparticle-induced biological effects such as inflammation and oxidative stress responses. Furthermore, this article underscored the point that the biokinetics of nanoparticles differ from those of larger particles and that the bioactivity and biokinetics of nanoparticles depend on several parameters, such as size, shape, chemistry, crystallinity, surface properties, agglomeration state, biopersistence, and dose. Sustained interest in the emerging area of nanotoxicology is indicated by the development of websites, international meetings, textbooks, and policy statements devoted to this topic over the last three years. The article by Oberdörster et al. has helped lay the scientific foundation for developing a multidisciplinary research program on nanotoxicology.

The second most cited article also concerns nanomaterials. “Manufactured Nanomaterials (Fullerenes, C_60_) Induce Oxidative Stress in the Brain of Juvenile Largemouth Bass” by Eva Oberdörster (Environ Health Perspect 112:1058–1062) has been cited an average of 34.6 times per year since it was published in July 2004. Oberdörster was the first to show that uncoated fullerenes can cause oxidative damage and depletion of glutathione *in vivo* in an aquatic species. The results of this research raise concerns about the potential for manufactured nanomaterials to cause adverse health effects in other species, including humans.

The third most cited *EHP* article is “Decrease in Anogenital Distance among Male Infants with Prenatal Phthalate Exposure” by Shanna H. Swan, Katharina M. Main, Fan Liu, Sara L. Stewart, Robin L. Kruse, Antonia M. Calafat, Catherine S. Mao, J. Bruce Redmon, Christine L. Ternand, Shannon Sullivan, J. Lynn Teague, and the Study for Future Families Research Team. This article, published in August 2005 (Environ Health Perspect 113:1056–1061), has been cited an average of 34.0 times per year. This study was the first to examine anogenital distance and other genital measurements in relation to prenatal exposure to phthalates in humans. This study was predicated on data from toxicologic studies in rodents showing that genital morphology can be altered by developmental exposure to phthalates, which may act through an antiandrogenic mechanism. Swan et al. found an association between male genital development and phthalate exposure, suggesting that male reproductive development in humans could be affected by prenatal exposure to environmentally relevant levels of phthalates.

The fourth most highly cited article is “Brominated Flame Retardants: Cause for Concern?” by Linda S. Birnbaum and Daniele F. Staskal. This review was published in January 2004 (Environ Health Perspect 112:9–17) and has been cited an average of 29.0 times per year. In this article, Birnbaum and Staskal discussed the scientific issues associated with the use of commercial mixtures of polybrominated diphenyl ethers, which are widely used as flame retardants. They provided an overview of the sources of these materials in the environment and discussed concerns related to the chemicals’ persistence, bioaccumulation, and potential for toxicity in humans. The authors also generated ideas and hypotheses concerning the potential mechanism(s) of action of these chemicals. Information gaps identified in this article have served as the basis for research by others in the field of environmental health sciences.

The timeliness of research on phthalates is underscored by the fact that the fifth most highly cited article is “Urinary Levels of Seven Phthalate Metabolites in the U.S. Population from the National Health and Nutrition Examination Survey (NHANES) 1999–2000” by Manori J. Silva, Dana B. Barr, John A. Reidy, Nicole A. Malek, Carolyn C. Hodge, Samuel P. Caudill, John W. Brock, Larry L. Needham, and Antonia M. Calafat. This paper was published in March 2004 (Environ Health Perspect112:331–338) and has been cited an average of 20.4 times per year. These researchers measured the urinary monoester metabolites of seven commonly used phthalates in more than 2,500 samples collected during 1999–2000 for selected demographic groups in the United States. The data support the conclusion that exposure to phthalates is widespread but varies according to age, sex, and ethnic group. Given the potential for human exposure in the United States, the study pointed to the need to evaluate the potential adverse health effects of phthalates at environmentally relevant levels.

*EHP* congratulates the authors of the most highly cited articles from the 2004–2008 period for their significant contribution to the environmental health science literature. These authors identified numerous gaps in our knowledge concerning exposure to and the potential adverse health effects of highly prevalent environmental pollutants such as phthalates, polybrominated diphenyl ethers, and nanomaterials. These highly cited articles have influenced the research agendas not only of individual researchers but also of agencies such as the U.S. Environmental Protection Agency, the Centers for Disease Control and Prevention, and the National Institute of Environmental Health Sciences.

## Figures and Tables

**Figure:**
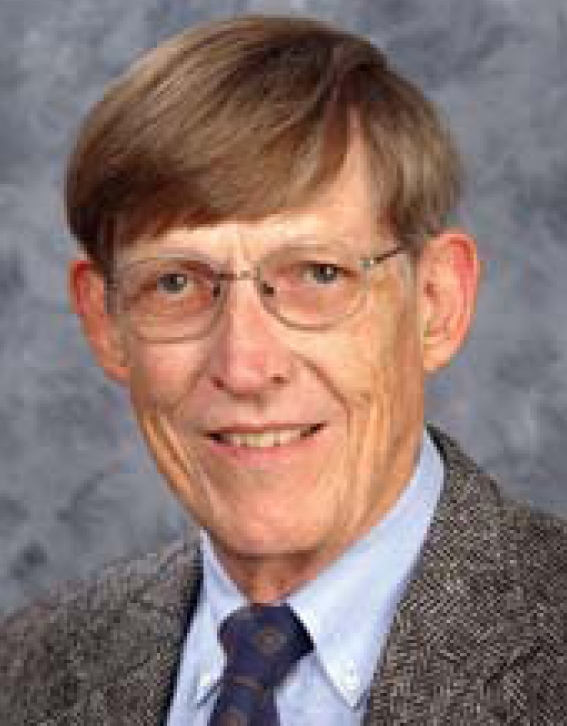
Hugh A. Tilson

